# The duration of nutrient limiting conditions can contribute to shaping subsequent diatom community composition: Insights from laboratory experiments

**DOI:** 10.1371/journal.pone.0333868

**Published:** 2026-07-21

**Authors:** Drajad S. Seto, Lee Karp-Boss, Mark L. Wells

**Affiliations:** 1 Department of Oceanography, School of Marine Sciences, University of Maine, Orono, Maine, United States of America; 2 Department of Cell and Molecular Biology, College of the Environment and Life Sciences, University of Rhode Island, Kingston, Rhode Island, United States of America; 3 Climate Change Institute, University of Maine, Orono, Maine, United States of America; NRSC: National Remote Sensing Centre, INDIA

## Abstract

Climate-driven increases in global surface water temperatures are enhancing upper ocean stratification and likely resulting in more prolonged periods of nutrient limitation. Although nutrient limitation in diatoms and their growth responses to increasing temperatures have been studied extensively, much less is known about their growth response to nutrient injection after prolonged durations of nutrient limitation. This study examines the growth response of three bloom-forming diatom species: *Pseudo-nitzschia pungens*, *P. australis*, and *Skeletonema costatum* after short-term (~2 week) and prolonged (~4 week) periods of nutrient limitation at five temperatures (9, 12, 15, 20, and 25°C). *Pseudo-nitzschia* species showed shorter lag times and higher specific growth rates than *S. costatum* after prolonged nutrient stress. These findings demonstrate that certain diatom species can exhibit faster growth recovery after prolonged nutrient limitation and in warmer conditions compared to others, providing new insights on drivers that shape phytoplankton communities.

## Introduction

Marine diatoms often dominate phytoplankton biomass in coastal and other high productivity regions of the oceans, particularly during spring blooms and upwelling events [1,2]. This is a highly diverse group of species that exhibit a wide range adaptive mechanisms for survival under short-term nutrient stress, including changes in photosynthetic efficiency [3], alteration in lipid composition [4], adjustment of sinking speeds [5], and altered metabolic pathways [6]. When nutrient stress progresses over longer time scales some species shift their metabolism to form resting spores as a strategy for survival [7–9]. This shift involves both metabolic and morphological changes that include the development of a thick silica coatings [7]. Cells emerge from these dormant stages and recover vegetative growth once conditions improve. Although not widely studied, the formation of resting stages has been only observed in centric diatom species so far [e.g., 7,10,11].

Not all diatoms form spores to survive nutrient stress. Some species, including certain pennate diatoms, appear to down-regulate or pause their growth stages in a type of “hibernation” that is not yet understood [9], while others may rely on nutrient storage or lower minimum quotas [12]. These divergent strategies suggest that growth responses of diatom to an injection of nutrients after prolonged starvation may differ, whereby species not substantially reorganizing their metabolic or morphological structures potentially could resume vegetative growth faster once conditions improve. The subsequent staggered lag times for re-initiating exponential growth might give an advantage to some species over others mediating succession during bloom formation. Although these community changes may be modulated by grazing [13], these grazing pressures typically occur well after community development in rapidly forming diatom blooms.

With continued ocean warming and the increase in the frequency, duration, and intensity of heat waves over the past decade [14], understanding the role of prolonged periods of seasonal stratification and nutrient limitation in shaping phytoplankton communities in costal ecosystems becomes even more relevant. While many studies have examined the effects of nutrient limitation and co-limitation on the physiology and ecology of phytoplankton, there is very little information about how the history of limitation (i.e., its duration) affects the recovery potential of a given species. Diatoms of the genus *Pseudo-nitzschia* in the coastal upwelling system of the California current have been often observed to dominate when upwelling is preceded by prolonged periods of abnormally warm conditions [15–17], but the nutritional history of the cells that generated these blooms is unknown and generally very difficult to assess in the field. Here, we conducted a laboratory study to examine how three coastal diatom species respond to macronutrient additions after Short-term (~12 days) and Prolonged (~27 days) periods of limitation; two species of *Pseudo-nitzschia*, a pennate genus that is not known to form resting spores, and one species of the genus *Skeletonema,* a centric genus that has the potential to produce resting spores in response to nutrient limitation [9,18]. We predicted that growth recovery from prolonged nutrient stress will differ between the two genera, with the two *Pseudo-nitzschia* species having a shorter lag time than *S. costatum*. We further examined if responses vary as a function of growth temperature to better understand the combined effects of warmer temperatures and nutrient limitation that cells may experience under prolonged periods of warming.

## Materials and methods

### Culture conditions

We used non-axenic cultures of three diatom species: toxigenic pennate diatom *Pseudo-nitzschia pungens* from the Gulf of Maine (EBB1, Peter Countway, Bigelow Laboratory, ME) and *P. australis* from coastal water off Washington State (Bryan D. Bill and Vera Trainer, NOAA), and a non-toxic centric diatom *Skeletonema costatum* strain (CCMP778; isolated from the Caribbean Sea in the North Atlantic). Cultures were grown in sterile autoclaved media that was prepared with filtered seawater (0.7 μm GF/F, Whatman^TM^, Pittsburg, PA, USA) collected from Frenchman Bay (ME, USA) or the University of Maine Darling Marine Center dock (Walpole, ME, USA). Filtered seawater was enriched with 16 µmol L^-1^ NO_3_^-^, 16 µmol L^-1^ (Si(OH)_4_, and 3 µmol L^-1^ PO_4_^3-^, conditions that reflect the deep water nutrient concentrations of the Gulf of Maine [19], along with 25% of L1 trace metals and vitamins concentrations [20]. Hereafter, we refer to the medium as “Gulf of Maine (GoM) media”. Cultures were maintained under cool, white fluorescent light (Philips TLD 36W/840, YZ36RL25) with a 14:10 h light:dark cycle.

### Experimental designs

Two independent experiments were conducted, with triplicate treatments. Experiment 1 aimed to determine the growth response of *P. pungens*, *P. australis* and *S. costatum* to short-term nutrient limitation (Short-term NL) and prolonged nutrient limitation (Prolonged NL) at a common sea surface temperature during spring/summer in the Gulf of Maine (16°C). Experiment 2 examined these limitation periods across five temperatures (9°C, 12°C, 15°C, 20°C, 25°C), reflecting Gulf of Maine seasonal norms and projected 2100 conditions [21]*.*

#### Experiment 1- Effect of nutrient limitation duration at 16°C.

**Fig 1:**
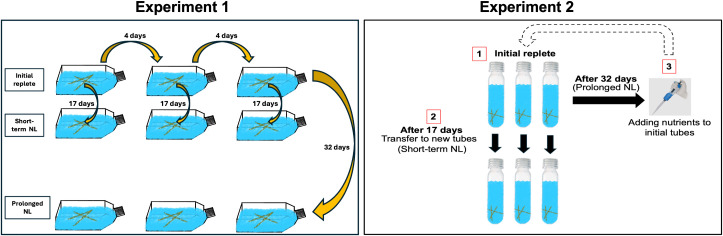
Experimental Design for Experiments 1 and 2. Cultures were grown under three conditions: Initial transfer into fresh, nutrient replete media, Short-term NL (17 days total, with effective limitation for ~12 days), and Prolonged NL (32 days total, with effective limitation for ~27 days). In Experiment 1, cultures were grown in 175 ml tissue culture flasks and transferred to fresh media after each limitation period. In Experiment 2, cultures were grown in 20 ml polycarbonate tubes across five temperatures (9°C, 12°C, 15°C, 20°C, 25°C); after Short-term NL, cultures were transferred to fresh media, while after Prolonged NL, nutrients were re-infused directly to the Initial culture tubes. Arrows represent the time (in days) between transfers to fresh media.

Cultures of *P. pungens*, *P. australis*, and *S. costatum* were grown in semi-continuous batch mode in 175 ml culture tissue flasks with GoM media at 16 ± 1°C (Fig 1). Cells were acclimated for > 10 generations before starting the experiment. Cells were considered ‘acclimated’ when growth rates varied less than 10% in consecutive butch cultures.

After acclimation, cells were transferred to fresh GoM media on day 0 of the experiment, termed “Initial replete” serving as the growth reference for statistical comparisons with Short-term and Prolonged NLs (Fig 1). In pre-liminary experiments we determined that nutrient concentrations for *P. pungens* had decreased to 2.59 µM for nitrate and 1.54 µM for silicate by day 4–5, while for *S. costatum*, the nutrient levels were below detection limit for nitrate and 0.92 µM for silicate (S3 Fig). The growth of both species reached stationary phase on day ~ 5. Cultures remained in stationary phase under the same light and temperature conditions for additional 12 days (i.e., a total of 17 days from the start of the experiment) to ensure nutrient depletion (hereafter, “Short-term NL”) or 32 days (“Prolonged NL”, ~ 27 days post-depletion) (Fig 1). While the stationary phase served as a practical indicator of nutrient limitation, this approach does not identify which specific nutrient(s) became limiting. Therefore, this study focuses on species-level recovery responses that were captured through specific growth rate (SGR) and lag time (LT) rather than on the physiological mechanisms underlying a particular nutrient deficiency. Growth trajectories during nutrient limitation and following transfers to fresh media are shown in Supplementary S4 Fig, indicating the timing of the stationary phase used to define Short-term and Prolonged NLs. A subset of these stationary cultures was transferred to fresh GOM media after these limitation periods, and sub-samples were taken daily and preserved with 1% Lugol’s iodine for cell counts. Exponential growth rates were determined by cell counts using a Sedgwick-Rafter chamber for enumeration under an inverted microscope (Nikon-TMS F). Growth experiments for *P. pungens* and *S. costatum* were conducted from July to September 2020; experiments with *P. australis* were conducted from May to July 2021; both experiments used the same basal seawater batch collected from the dock of the University of Maine Darling Marine Center.

#### Experiment 2– Interactive effect of nutrient limitation duration and temperature.

The same initial experimental plan was used for Experiment 2. Cultures were grown in triplicate 28 ml polycarbonate tubes (Nalgene^TM^ Oak Ridge Centrifuge Tube) with 20 ml GoM media at 9°C, 12°C, 15°C, 20°C, and 25°C (Firstek, TG-5 model, Taiwan). After acclimation, cells were transferred at day 0 and the experiment followed by the same protocol above. For Short-term NL, 1 ml of each replicate was inoculated into fresh GoM media, and growth was tracked for ~10 days. For Prolonged limitation, nutrients (16 µmol L^-1^ NO_3_^-^, 16 µmol L^-1^ (Si(OH)_4_, and 3 µmol L^-1^ PO_4_^3-^) and 25% of L1 trace metals and vitamins concentrations [20] were added directly to initial cultures to avoid diluting cell abundances below detection. Because of the multi-factorial nature of the experiment (with replications), manual cell counts were too time consuming, and we used the faster chlorophyll fluorescence approach (Turner Design, USA, model 10-AU-005-CE) [22] to estimate growth rates, keeping in mind biases associated with this method. We validated chlorophyll fluorescence measurements with cell counts for all species at 16°C across Initial replete, Short-term NL, and Prolonged NL conditions. Due to the exponential nature of cell growth and non-linear fluorescence responses, log 2-transformed cell counts were plotted against log2-transformed fluorescence units, yielding strong overall correlations (r^2^ = 0.97 for *P. pungens*, r^2^ = 0.88 for *S. costatum,* r^2^ = 0.87 for *P. australis*); condition-specific correlations are reported in S1 Table, with plots for the Initial replete, Short-term, and Prolonged NLs in S1 Fig. Growth response experiments for *P. pungens* and *S. costatum* were conducted from December 2020 to February 2021 using Frenchman Bay filtered (GF/F) seawater as a basal media, while growth experiments with *P. australis* were conducted from May to July 2021 using filtered seawater from the dock of the University of Maine Darling Marine Center.

### Growth measurements

Specific growth rates (SGR) were calculated as:


$$\begin{array}{c} SGR=\frac{ln(\frac{Nt}{N\text{0}})}{\Delta t} \end{array}$$ (1)

Where N_t_ and N_0_ are cell concentrations (Experiment 1) or fluorescence-derived biomass (Experiment 2) at time t and initial time, derived from the exponential phase [22]. For each replicate, N_0_ and N_t_ were measured individually. Lag times (LT) were defined as the number of days from nutrient resupply (day 0) until the onset of the exponential growth phase, identified as the point where cell counts (Experiment 1) or in vivo fluorescence (Experiment 2) began a consistent logarithmic increase, determined by fitting an exponential growth model to daily measurements [22]. If no exponential growth occurred within the experimental timeframe (e.g., ~10 days), LT was recorded as not applicable (NA). SGR and LT are used here as integrative measures of physiological recovery capacity following nutrient stress, independent of the identity of the limiting nutrient. All replicate SGR and LT values for Experiments 1 and 2 are reported in the Supporting Information (Table S2).

### Statistical analysis

For Experiment 1, two-way ANOVA tested the effects of species (*P. pungens*, *P. australis*, *S. costatum*) and duration (Initial replete, Short-term NL, and Prolonged NL) on SGR and LT, using triplicate data. Tukey’s HSD for post-hoc tests identified specific differences across all levels of duration: for SGR, species differences were examined within each duration (e.g., *P. pungens* vs. *S. costatum* in Initial replete, Short-term and Prolonged NLs) and duration differences within each species (e.g., Short-term NL vs. Initial replete for *P. pungens*); for LT, duration differences were examined within each species (e.g., Short-term NL vs. Initial replete for *P. pungens*), and species differences were assessed within each species (e.g., *P. australis* vs. *P. pungens* in Prolonged NL).

For Experiment 2, two separate analyses were conducted using triplicate data: 1) temperature and 2) duration effects. For temperature effects, one-way ANOVA tested the effect of temperature (9°C, 12°C, 15°C, 20°C, 25°C) on SGR and LT within each species and condition (Initial replete, Short-term and Prolonged NLs). Tukey’s HSD post-hoc tests examined significant differences from 15°C (ambient/control) within each species and condition. For duration effects, two-way ANOVA tested the effects of species and duration within each temperature on SGR and LT. Tukey’s HSD post-hoc testes examined significant differences from Initial replete conditions within each species and temperature. Analyses were performed in R version 2024.09.1+394.

## Results

### Experiment 1 – Effect of nutrient limitation duration at 16°C

**Fig 2:**
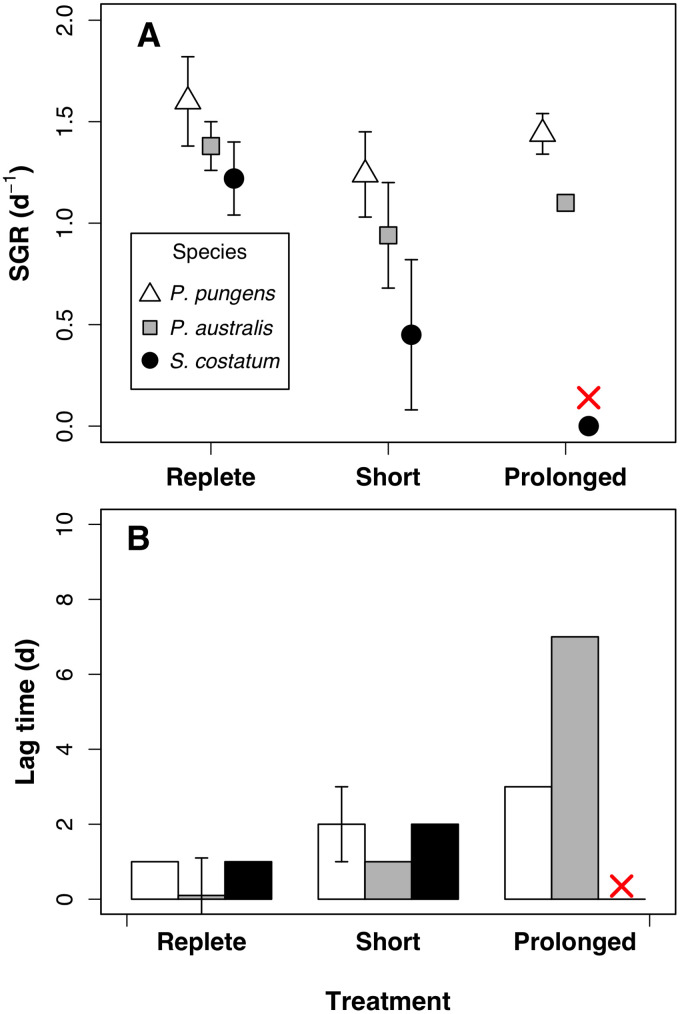
Experiment 1 results. Means and standard deviations of SGR (panel A) and LT (Panel B) of *P. pungens* (white triangle/bar), *P. australis* (grey square/bar), and *S. costatum* (black circle/bar) in the Initial replete nutrient treatment, and after Short-term and Prolonged NLs. Note that the *P. australis* treatment has one “replicate” (n = 1) in the prolonged treatment.

Under Initial replete conditions, all species showed rapid growth with minimal LT (Table 1, Figs 2A, 2B). After exposure to Short-term NL, all species recovered upon nutrient resupply, with *P. pungens* maintaining a higher SGR than *S. costatum* (p < 0.01, Tukey’s HSD); *P. australis* showed intermediate SGR, yet significantly higher than *S. costatum* (p < 0.05). LT increased significantly for *P. pungens* and *S. costatum* (p < 0.05 vs. Initial replete) but remained unchanged for *P. australis* (p > 0.05). Following Prolonged NL, *P. pungens* and *P. australis* maintained high SGRs compared to *S. costatum*, which showed no growth (p < 0.001) within five days period. LT further increased for *P. pungens* (p < 0.001 vs. Initial replete) and *P. australis* (not statistically tested vs. Initial replete due to insufficient replicates).

**Table 1:** Specific growth rates (SGR) and lag times (LT) for three diatom species under varying nutrient limitation durations at 16°C.

Species	Condition	Temp. (°C)	SGR (Mean ± SD, d^-1^)	LT (Mean ± SD, days)
*P. pungens*	Initial replete	16	1.60 ± 0.22	1 ± 0^*^
*P. pungens*	Short-term NL	16	1.24 ± 0.21^*†^	2 ± 1^†^
*P. pungens*	Prolonged NL	16	1.44 ± 0.1^*^	3 ± 0^†^
*P. australis*	Initial replete	16	1.38 ± 0.12	0 ± 1
*P. australis*	Short-term NL	16	0.94 ± 0.26^†^	1 ± 0
*P. australis*	Prolonged NL	16	1.10 (n = 1)	7 (n = 1)
*S. costatum*	Initial replete	16	1.22 ± 0.18	1 ± 0^*^
*S. costatum*	Short-term NL	16	0.45 ± 0.37^†^	2 ± 0^†^
*S. costatum*	Prolonged NL	16	0^†^	NA

NA indicates no growth observed. LT error is ± 1 day due to daily sampling. *P. australis* prolonged reflects n = 1 and is excluded from species difference comparisons due to insufficient replicates. Means and standard deviation are calculated from triplicate data (except where noted). Asterisks (*) indicate significant changes in species differences (relative to Initial replete) across durations (p < 0.05, Tukey’s HSD). Dagger (†) indicate significant differences from Initial replete within each species (p < 0.05, Tukey’s HSD).

Two-way ANOVA indicated significant effects of species (F(2,16) = 38.75, p < 0.001), limitation duration (F(2,16) = 20.35, p < 0.001), and their interaction (F(4,16)= 5.32, p < 0.01) on SGR (Fig 2A). Tukey’s HSD showed that the SGR of *P. pungens* was significantly higher than *S. costatum* in Short-term NL (p < 0.001) and Prolonged NL (p < 0.0001) compared to Initial replete growth conditions; *P. australis* in Prolonged NL (n = 1) was not included in species difference comparisons due to insufficient replicates. Within species, Short-term and Prolonged NLs SGRs were significantly lower than Initial replete growth conditions for *S. costatum* (p < 0.01) but not significant for *P. pungens* and *P. australis* (p > 0.05). For LT (excluding *S. costatum* prolonged due to no growth, as LT could not be measured), the main effect of species was not significant (F(2,14) = 2.10, p > 0.05), but duration (F(2,14) = 59.72, p < 0.001), and their interaction (F(3,14) = 21.62, p < 0.001) were significant (Fig 2B). Tukey’s HSD showed that *P. australis* had the shortest LT among the three species, under Initial replete growth conditions. The LTs in Short-term NL experiments were higher than Initial replete conditions for *P. pungens* and *S. costatum* (not significant for *P. pungens*; significant for *S. costatum*, p < 0.05). *P. pungens* also showed a longer LT in Prolonged NL experiments (p < 0.001). For *P. australis*, Short-term NL LT was not significantly higher than Initial replete growth conditions (p > 0.05), and Prolonged NL (n = 1) is reported descriptively.

### Experiment 2 – Interactive effects of nutrient limitation duration and temperature

**Fig 3:**
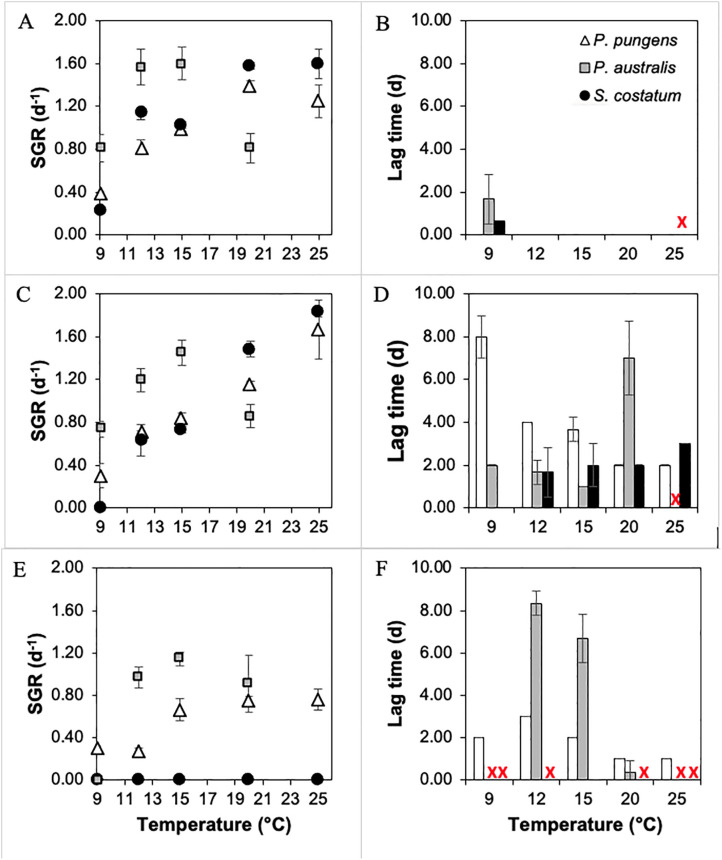
Experimental 2 results. Mean and standard deviations of SGR and LT as a function of the temperatures of *P. pungens* (white triangle/bar), *P. australis* (grey square/bar), and *S. costatum* (black circle/bar) in Initial replete (A&B) (n = 3), Short term NL (C&D) (n = 3), and Prolonged NL (E&F) (n = 3). SGR were determined from the regression slope on increasing in-vivo fluorescence during the exponential phase.

Under nutrient initial replete conditions, the SGR increased with temperature for all species, although the temperature optima varied (Fig 3A, Table 2). *P. pungens* SGR increased from 0.38 ± 0.02 d^-1^ at 9°C to 1.40 ± 0.04 d^-1^ at 20°C (F(4,10) = 81.1, p < 0.001, one-way ANOVA), declining to 1.25 ± 0.15 d^-1^ at 25°C (p < 0.05 vs. 15°C), with no LT observed across the range of 9−25°C (Fig 3B). *P. australis* reached highest SGR at 15°C (1.60 ± 0.15 d^-1^, F(4,10) =74.8, p < 0.001), dropping to 0 d^-1^ at 25°C (p < 0.001 vs. 15°C), with a LT of 2 ± 1 days at 9°C (p < 0.001 vs 15°C). *S. costatum* SGR increased from 0.23 ± 0.04 d^-1^ at 9°C to 1.6 ± 0.14 d^-1^ at 25°C (F(4,10) = 182.1, p < 0.001), with a 2 ± 1 day LT at 9°C (p < 0.001 vs 15°C).

**Table 2:** Specific growth rate (SGR) and lag time (LT) for three diatom species under varying nutrient limitation duration and at different temperatures.

Species	Condition	Temp. (°C)	SGR (Mean ± SD, d^-1^)	LT (Mean ± SD, day)
*P. pungens*	Initial Replete	9	0.38 ± 0.02*	0 ± 0
*P. pungens*	Initial Replete	12	0.82 ± 0.07	0 ± 0
*P. pungens*	Initial Replete	15	0.99 ± 0.02	0 ± 0
*P. pungens*	Initial Replete	20	1.40 ± 0.04*	0 ± 0
*P. pungens*	Initial Replete	25	1.25 ± 0.15*	0 ± 0
*P. pungens*	Short-term NL	9	0.3 ± 0.12*	8 ± 1*†
*P. pungens*	Short-term NL	12	0.71 ± 0.01†	4 ± 0†
*P. pungens*	Short-term NL	15	0.84 ± 0.05	4 ± 1†
*P. pungens*	Short-term NL	20	1.15 ± 0.04†	2 ± 0*†
*P. pungens*	Short-term NL	25	1.67 ± 0.28*†	2 ± 0*†
*P. pungens*	Prolonged NL	9	0.30 ± 0.02*	2 ± 0†
*P. pungens*	Prolonged NL	12	0.26 ± 0.04*†	3 ± 0*†
*P. pungens*	Prolonged NL	15	0.66 ± 0.10†	2 ± 0†
*P. pungens*	Prolonged NL	20	0.75 ± 0.04†	1 ± 0*†
*P. pungens*	Prolonged NL	25	0.76 ± 0.1†	1 ± 0*†
*P. australis*	Initial Replete	9	0.81 ± 0.13*	2 ± 1*
*P. australis*	Initial Replete	12	1.57 ± 0.17	0 ± 0
*P. australis*	Initial Replete	15	1.60 ± 0.15	0 ± 0
*P. australis*	Initial Replete	20	0.81 ± 0.14*	0 ± 0
*P. australis*	Initial Replete	25	–	–
*P. australis*	Short-term NL	9	0.74 ± 0.07*	2 ± 0
*P. australis*	Short-term NL	12	1.20 ± 0.11†	2 ± 1†
*P. australis*	Short-term NL	15	1.45 ± 0.12	1 ± 0†
*P. australis*	Short-term NL	20	0.86 ± 0.11*†	7 ± 2*†
*P. australis*	Short-term NL	25	–	–
*P. australis*	Prolonged NL	9	0.00 ± 0.00*†	NA
*P. australis*	Prolonged NL	12	1.05 ± 0.15†	8 ± 1†
*P. australis*	Prolonged NL	15	1.20 ± 0.10†	7 ± 1†
*P. australis*	Prolonged NL	20	0.87 ± 0.20	1 ± 1*
*P. australis*	Prolonged NL	25	–	–
*S. costatum*	Initial Replete	9	0.23 ± 0.04*	1 ± 1
*S. costatum*	Initial Replete	12	1.13 ± 0.05	0 ± 0
*S. costatum*	Initial Replete	15	1.03 ± 0.02	0 ± 0
*S. costatum*	Initial Replete	20	1.58 ± 0.03*	0 ± 0
*S. costatum*	Initial Replete	25	1.60 ± 0.14*	0 ± 0
*S. costatum*	Short-term NL	9	0.00 ± 0.00*†	NA
*S. costatum*	Short-term NL	12	0.63 ± 0.15†	2 ± 1
*S. costatum*	Short-term NL	15	0.73 ± 0.03†	2 ± 1†
*S. costatum*	Short-term NL	20	1.49 ± 0.08*	2 ± 0†
*S. costatum*	Short-term NL	25	1.83 ± 0.05*	3 ± 0†
*S. costatum*	Prolonged NL	9	0.00 ± 0.00*†	NA
*S. costatum*	Prolonged NL	12	0.00 ± 0.00*†	NA
*S. costatum*	Prolonged NL	15	0.00 ± 0.00*†	NA
*S. costatum*	Prolonged NL	20	0.00 ± 0.00*†	NA
*S. costatum*	Prolonged NL	25	0.00 ± 0.00*†	NA

NA indicates no growth observed. LT error is ± 1 day due to daily sampling. Means and standard deviations are calculated from triplicate data. Asterisks (*) indicate significant differences from 15°C (ambient/control) within each species and condition (p < 0.05, Tukey’s HSD, one-way ANOVA for temperature effects). Daggers (†) indicate significant differences from Initial replete growth condition within each species and temperature (p < 0.05, Tukey’s HSD, two-way ANOVA for species and duration effects).

After Short-term NL, the SGR of *P. pungens* also increased from 0.30 ± 0.12 d^-1^ at 9°C to 1.67 ± 0.28 d^-1^ at 25°C (F(4.10) = 41.3, p < 0.001), with a longer LT at 9°C (8 ± 1 days, p < 0.001 vs 15°C, Fig 3D). *P. australis* peaked at 15°C (1.45 ± 0.12 d^-1^, F(4,10) = 106.2, p < 0.001), but showed no growth at 25°C (0.00 ± 0.00 d^-1^, p < 0.001 vs. 15°C) across all conditions, as cells failed to grow in the Initial replete conditions at this temperature (Table 2); LT increased to 7 ± 2 days at 20°C (p < 0.001 vs. 15°C). *S. costatum* did not grow at 9°C and had a max SGR of 1.83 ± 0.05 d^-1^ at 25°C (F(4,10) = 256.8, p < 0.001), with LT of 2–3 days (Fig 3C & 3D).

Growth responses of these species were markedly different after prolonged nutrient limitation. *P. pungens* SGR increased from 0.30 ± 0.02 d^-1^ at 9°C to 0.76 ± 0.10 d^-1^ at 20–25°C (F(4,10) = 38.5, p < 0.001), with short LT of 1–3 days (Fig 3E, 3F). *P. australis* displayed no detectable growth at 9°C, peaking the SGR at 15°C (1.20 ± 0.10 d^-1^, F(4,10) = 69.5.4, p < 0.001), with longer LT of 8 ± 1 days at 12–15°C (p > 0.05, 12°C vs. 15°C), 1 ± 0 days at 20°C (p < 0.001 vs 15°C). As observed in Experiment 1, *S. costatum* did not respond well to prolonged nutrient limitation.

Within each temperature, treatment nutrient limitation duration significantly affected SGR and LT (Table 2). For example, compared to Initial replete growth conditions at 15°C, SGR of *P. pungens* decreased from 0.99 ± 0.02 d^-1^ to 0.66 ± 0.10 d^-1^ in Prolonged NL treatment (p < 0.01) and LT increased for *P. australis* (from no LT to 7 ± 1 days; p < 0.001). At 20°C, *P. australis* showed an increase in LT in Short-term NL increased (7 ± 2 days) compared to Initial replete growth conditions (0 ± 0 days, p < 0.001), while *S. costatum* SGR did not resume growth after Prolonged NL (during the window of observation) compared to Initial replete (1.58 ± 0.07 d^-1^, p < 0.001). These patterns show the combined influence of nutrient limitation duration and temperature on diatom responses (Table 2, Fig 3).

## Discussion

Increasing surface water temperatures have direct effects on cell metabolism, and indirect effects due to the increase in upper ocean stratification and hence the availability of nutrients to phytoplankton [23,24]. While there is a rich literature on the direct effects of changing temperature and nutrient availability on diatoms [e.g., 25–27], far less is known about how the duration of nutrient depletion associated with warming surface waters might influence diatom metabolism. We examine here whether the duration of nutrient stress may become a selective pressure influencing the composition of diatom communities, and how outcomes may vary under different growth temperatures.

The duration of nutrient limitation significantly impacted the ability of these diatoms to recover after nutrient stress in our experiments. All three species recovered growth upon nutrient reinfusion after a short period of nutrient limitation (~12 days) (Figs 2A, 3C), though LT varied with temperature (Figs 2B, 3D). However, these responses diverged after prolonged (~27 days) nutrient limitation. While both *Pseudo-nitzschia* species quickly responded to the nutrient additions, *S. costatum* was unable to recover its growth under these conditions over the duration of the experiment. Remarkably, LT for *P. pungens* increased by only 1−3 days (relative to Initial replete growth conditions) when nutrients were added after 27 days of depletion (Experiment 1 & 2; Figs 2A & 3F). The resultant growth rates of both *Pseudo-nitzschia* species were somewhat lower relative to the Initial replete growth conditions but still remained high (0.8 d^-1^ vs ~ 1.5 d^-1^ for cells transferred to fresh media every 4 days; Table 1; Experiment 1, Fig 2A). This rapid response was observed over the range of temperatures tested (Fig 3E). The implication of these findings is that *S. costatum* would have a low probability of comprising a significant portion of the natural blooming phytoplankton assemblage after long periods of nutrient stress end through upwelling or enhanced mixing, whereas both *P. pungens* and *P. australis* likely could rapidly flourish under these conditions.

We cannot attribute a specific cause for these different growth responses in our experiments. The lack of growth recovery in *S. costatum* could reflect cell death, resting spore formation, or a reversible dormant state. While cells were examined microscopically, no clear resting spore morphology was observed, and we cannot conclusively distinguish among these possible states. However, other internal cellular mechanisms also could have been at play. For example, the intracellular levels of sterol sulfates (Sts) associated with cell senescence increase as *Skeletonema* cells age, linked to an apoptosis-like death mechanism [28]. Alternatively, or perhaps in conjunction, nutrient stress of the genus *Skeletonema* has been shown to result in the over-production of reactive oxygen species (ROS), causing oxidative damage to cellular components [29]. Regardless of the specific mechanism, the results demonstrate that the *S. costatum* strain tested here is poorly adapted for a rapid shift to the growth phase after prolonged nutrient stress when all other conditions are suitable for rapid growth.

The effect of temperature on this response also varied among the three diatom species but in a different way. Both *P. pungens* and *S. costatum* increased their SGR with increasing temperature, consistent with previous studies [e.g., 30,31]. Short-term NL did not alter this pattern (Fig 3A, 3C). After prolonged nutrient stress, *P. pungens* exhibited a rapid growth response, but SGR decreased by up to ~50% at all temperatures relative to the Initial replete growth conditions (Fig 3E**)**. So, while the LT were short, prolonged nutrient depletion still impeded the metabolic functioning of *P. pungens* to some degree.

In contrast to both *P. pungens* and *S. costatum*, SGR of *P. australis* decreased at the highest temperature (25°C) (Fig 3A, 3C, 3E**)**, in agreement with previous findings [e.g., 17,32]. Even so, its SGR after prolonged NL at 20°C was nearly identical to that under the initial replete conditions, and its LT was even shorter (Fig 3E, 3F**)** relative to Short-term NL (Fig 3C, 3D**)**. In other words, the findings indicate that this strain of *P. australis* can flourish after Prolonged NL at ≤ 20°C; i.e., ambient temperatures in most temperate coastal and offshore upwelling regimes.

While nutrient concentrations in cultures were not measured routinely in this study, our preliminary experiments confirm that nitrate was entirely depleted in *S. costatum* cultures by day 5. Similarly, nitrate declined in the *P. pungens* cultures from 19.11 µM at inoculation to 2.59 µM by day 5, while cell abundance increased from 333 to 18,083 cells mL^-1^. This corresponds to a drawdown of 16.52 µM nitrate, which, when divided by the increase in cell number (1.775x10^7^ cells L^-1^), implies ~0.93 pmol N assimilated per cell. This estimate is of the same order of magnitude as quotas reported quotas for smaller *Pseudo-nitzschia* species such as *P. subcurvata* (0.27–0.38 pmol N cell^-1^ [33]) and since *P. pungens* typically has a larger cell size compared to *P. subcurvata*, its nitrogen quota are likely correspondingly higher. Given that only ~2.6 µM nitrate remained by day 5, nitrate limitation would have occurred shortly afterwards, as confirmed by the co-occurrence of senescence.

One could argue that factors other than nutrients, such as accumulation of toxic metabolic byproducts (e.g., oxylipins) or shifts in the associated microbial community, could have led to the senescence of cultures, as is often observed in F/2 or L1 media where growth reaches senescence before nutrients are fully depleted. Because the initial nutrient concentration in the GoM media were significantly lower than F/2 or L1, and because cultures transferred to nutrient replete media on day 17 (or 32) would also be diluted, such transfer potentially may have relieved stressors other than nutrient limitation. However, in Experiment 2 under the Prolonged treatment, nutrients were directly injected to culture flasks (no transfer and dilution), so the observed responses represent the release from nutrient limitation and not the removal of growth inhibiting factors. The consistent patterns observed from both Experiment 1 (at 16°C) and Experiment 2 (with the 15^o^C incubation), despite slight differences in methodology (transfer vs injection of nutrients), support that nutrient limitation in these cultures was essentially complete shortly after day 5 of the experiments.

Further studies are needed to develop a mechanistic understanding of the underlying metabolic processes during short-term and prolonged periods of nutrient limitations across a broader range of taxa. Nevertheless, the findings from this study provide initial support for the idea that the duration of nutrient limitation can play a role in shaping phytoplankton communities after re-supply of nutrients; a factor that is not currently considered in models such as the phytoplankton Darwin model [e.g., 34,35]. The implication is that the development and progression of bloom assemblages may be strongly influenced across a broader timeline of co-interacting bottom-up, beginning far before bloom initiation.

In the absence of selective grazing pressures, taxa that are ready to resume growth immediately upon nutrient re-supply may be able to temporarily escape grazing control to initially dominate the community. Notably, field observations indicate that prolonged warming events are linked to the onset of *Pseudo-nitzschia* blooms along the West Coast of the United States. Perhaps the best example is the massive *Pseudo-nitzschia* bloom along much of the Western coast of N. America in 2015 [17,36]. Anomalously warm (nutrient-depleted) waters were advected into the coastal region (the “warm blob”) in three winter months prior to the onset of seasonal upwelling. This upwelling created an intense, spatially continuous nearly monospecific *Pseudo-nitzschia* bloom for much of the western shore of N. America [17,36]. Furthermore, on the opposite coast, anomalously warm and drought summer conditions (i.e., low nutrient influx from riverine flow or vertical mixing) in the Gulf of Maine region during 2016 preceded the first recorded, and spatially extensive toxic *Pseudo-nitzschia* bloom. Unlike the 2015 bloom off the west coast, this bloom happened during the fall turnover in 2016, replacing the more diverse species composition that normally is observed [15]. These natural blooms, showing that members of the genus *Pseudo-nitzschia* can quickly resume growth even after experiencing nutrient limitation for about one month, are consistent with growth responses of *Pseudo-nitzschia* in our experiments. Results from this study provide new insights into mechanisms by which anomalous warming and prolonged nutrient limitation can potentially modulate the structure of diatom assemblages in coastal waters.

## Conclusion

The findings here show the extraordinary ability of two *Pseudo-nitzschia* spp. to quickly enter exponential growth after a prolonged nutrient limitation, in contrast to the *Skeletonema* sp. tested here. Although only three diatom species were tested here due to the intensive effort required for these long-duration experiments, these results provide an important foundation for future broader comparisons. As these experiments used non-axenic cultures, associated bacterial communities were present and may have contributed some variability to the observed responses. However, the laboratory setting, while differing in many ways from ocean waters, captures ecologically relevant dynamics consistent with field observation, supporting the idea that the nutrient stress duration is potentially a vital driver regulating natural diatom assemblages during at least the early stages of bloom development. The implication is that it is important to consider the nutritional history of species when evaluating their fitness to a changing environment. It is noteworthy that *Pseudo-nitzschia* species dominated the phytoplankton response in all mesoscale iron-enrichment experiments in High Nitrate Low Chlorophyll (HNLC) regions [37]. A better understanding of the different responses among diatoms after prolonged nutrient stress might come from transcriptomic experiments to elucidate the underlying cellular mechanisms. This work introduces a conceptual framework for how ocean warming may affect the timing and potential occurrence of blooms dominated by specific taxa in coastal and oceanic waters.

## Supporting information

S1 Fig Validation of fluorescence proxy using log2 cell count vs log2 fluorescence unit for three diatom species. (TIF)

S2 Fig Microscopic images *P. pungens* and *S. costatum* after Prolonged NL at 16°C in Experiment 1. (TIF)

S3 Fig Macronutrients drawdown for *P. pungens* and *S. costatum.* (TIF)

S4 Fig Growth over time for *P. pungens* and *S. costatum.* (TIF)

S1 Table Condition-specific correlation between log2 cell counts and log2 fluorescence unit for three diatom species in Experiment 1. (DOCX)

S2 Table All the specific growth rate (SGR) and lag time (LT) data from experiments 1 and 2. (XLS)
