# Supplementary material for: The duration of nutrient limiting conditions can contribute to shaping subsequent diatom community composition: Insights from laboratory experiments
Source: PLoS One. 2026 Jul 21;21(7):e0333868. doi: 10.1371/journal.pone.0333868 (PMC13387546; doi:10.1371/journal.pone.0333868)
Supplement: S1 Table — (DOCX) [file pone.0333868.s005.docx]

**Table S1. Condition-specific correlation between log2 cell counts and log2 fluorescence unit for three diatom species in Experiment 1**

| **Species** | **Condition** | **Temperature (°C)** | **R^2^ (Log2 Cell Number vs. Log2 FU)** |
| --- | --- | --- | --- |
| *P. pungens* | Initial | 16 | 0.99 |
| *P. pungens* | Short NL | 16 | 0.99 |
| *P. pungens* | Prolonged NL | 16 | 0.94 |
| *P. australis* | Initial | 16 | 0.87 |
| *P. australis* | Short NL | 16 | 0.87 |
| *P. australis* | Prolonged NL | 16 | 0.81 (n=1) |
| *S. costatum* | Initial | 16 | 0.88 |
| *S. costatum* | Short NL | 16 | 0.88 |
| *S. costatum* | Prolonged NL | 16 | NA (no growth) |
