# Supplementary material for: The duration of nutrient limiting conditions can contribute to shaping subsequent diatom community composition: Insights from laboratory experiments
Source: PLoS One. 2026 Jul 21;21(7):e0333868. doi: 10.1371/journal.pone.0333868 (PMC13387546; doi:10.1371/journal.pone.0333868)
Supplement: Attachment — Submitted filename: Response to reviewer (academic editor)_Seto et al.docx [file pone.0333868.s008.docx]

December 22, 2025

Dr. Rajdeep Roy

PLOS One Academic Editor

Re: The duration of nutrient limiting conditions can contribute to shaping subsequent diatom community composition; insight from laboratory experiments

Dear Dr. Roy,

We would like to thank you for the time you have taken to handle our paper and provide your feedback. We were surprised, however, by your decision to reject the manuscript given the very positive feedback from reviewer 1, who stated that “This research is fascinating, I’m happy to have the opportunity to review. It’s an excellent manuscript that contributes meaningfully to our understanding of diatom ecology and nutrient limitation effects, great work!”. In your evaluation, we believe there is some misunderstanding about the structure of the experiments, which may require some text revision to clarify what was done. We explain using your comments.

**1. “The authors suggest nutrient depletion from 16 µmol of nitrate and silicate to < 2 µmol within 5 days and then culture remains in that condition for next 12 days. This is very intriguing how only ambient temperature support growth for next 2 weeks without any nutrients”**

You are correct that there would be no growth after the nutrients were consumed, and indeed that is the case. As we state in the manuscript (line 138), the cultures reached a stationary phase after 5 days, the stage where cell growth ceases but cells do not die. We go on to explain that cells remained in stationary phase for the following 12 days. Due to space considerations, we did not include a figure of cell numbers/abundance over time, but perhaps it would be helpful to include this graph as a supplementary figure.

We note that this behavior (growth followed by an extended period of senescence), is very common with phytoplankton species. Indeed diatom cultures are known to survive in stationary phase for weeks (e.g., Berges and Falkowski 1998; Handy et al. 2024, and see below).The mechanisms underlying this behavior, and the potential for subsequent recovery of growth, are little understood and is a key focus of this study.

The cells in our experiments resumed growth only when nutrients were re-introduced after this 12-day period of stationary phase, ‘mimicking’, for example, the re-introduction of nutrients to nutrient-depleted surface waters by vertical mixing. This response also is not unexpected, and indeed all three cultures showed robust growth after nutrient introduction after this “short” period of senescence. What is unique in our study, according to our literature searches, is that we tested how effectively growth re-initiates after a much longer period in stationary phase (27 days), and whether there were differences in this response among the three diatom species studied. The robust response of two, but not the other, is the important finding here; one which conceptually may explain why blooms comprise different diatoms at different times; a major conundrum in biological oceanography.

2. “**The data presented do not show much difference between initial growth phase and days thereafter under nutrient deplete condition. This cannot happen!”**

This misunderstanding stems from the misconception above. The experiments did not compare the initial growth rates with growth under the nutrient deplete (stationary phase) conditions. We compare instead the initial growth rates with those immediately after the nutrient ‘starved’ cells in the stationary phase were transferred to new fresh media or received a new injection of nutrients (i.e. after 12 or 27 days of nutrient depletion). The results show that when nutrients are resupplied after short ‘starvations’ (12 days) all three species resumed growth similar to that observed initially, but when experiencing a longer-term ‘starvations’ (27 days) the *Skeletonema* sp. did not grow upon the re-introduction of nutrients but the *Pseudo-nitzschia* spp. responded as if there had been no nutrient stress at all (i.e., growth rates and lag times were very similar to the growth response on Day 0 (i.e., with cells taken from rapidly growing seed cultures). In other words, some species of the *Pseudo-nitzschia* genus (tested here) can cease to grow when starved for nutrients but their metabolic ‘machinery’ is ready to capitalize on nutrients as soon as they become available. In contrast, the *Skeletonema* sp. here lost this ability and would require more time to recover. This difference is highly relevant to understanding the relative global success of *Pseudo-nitzschia* species.

3. **“Even, some of the growth rates measured are higher than measured growth rates of Diatoms (See some of the publications by Victor Smetecek and others from natural environment and experimental set up)”**

You are correct that the diatom growth rates here are higher than in some other studies, and relative to some estimates of in-situ rates, however, the growth rates in our experiments are not unusual. There are many publications showing diatom growth rates in laboratory cultures being > 1 per day, and indeed the growth rates in our experiments lie within the range reported in the literature for laboratory cultures of *Pseudo-nitzschia* (e.g., McCabe et al. 2016; Pednekar et al. 2018) and *Skeletonema* (e.g., Kaeriyama et al. 2011; Li et al. 2021). It is more difficult to directly compare growth rates under “optimal” laboratory conditions with those in-situ, where non-nutrient growth limitations influence the production and accumulation rates of cells (e.g., light limitation due to shorter days and mixing to depth, and grazing pressures that limit cell accumulation rates). Even so, the growth rates measured in these experiments also fall with the range of those observed for phytoplankton in natural marine water (e.g., the excellent review paper of Laws 2013. Evaluation of in situ phytoplankton growth rates: a synthesis of data from varied approaches” Annu. Rev. Mar. Sci.5:247-68). So, there is nothing suspect about the growth rates we report in our findings.

On the other side of things, we realize that we did make an error with the data provided in Supplementary material table 1 (S1_Table). Especially, the growth rate values for *Pseudo-nitzschia pungens* under short-term nutrient limitation at 9°C and 12°C were inadvertently swapped due to an input error in the R script used for the statistical analysis. We appreciate the editor’s careful attention in identifying this issue. The growth rate values shown in the main manuscript figures are correct and unaffected, as they generated independently from the original Excel dataset rather than the R script output. Further, we made only minor adjustment to the results section after this reanalysis, which can be tracked from the track change. To ensure full transparency and facilitate reanalysis, we have now provided all raw growth rate data in the supplementary materials (S3_Table). Additionally, as we note above, we probably also should have provided a supplementary figure showing cell abundance over time in the cultures, which would help clarify the culture behavior between the nutrient re-introductions (S5_Fig). However, we do not believe this presentation issues negate the importance of the work, as interpreted by Reviewer 1, or should result in outright rejection of the manuscript for publication. We respectfully request that the editor re-examine this decision, and we welcome their help to ensure that we optimize the revisions.

Sincerely,

Drajad Seto

(on behalf of all authors).

**References**

Berges, J. A., and P. G. Falkowski. 1998. Physiological stress and cell death in marine phytoplankton: induction of proteases in response to nitrogen or light limitation. Limnology and Oceanography **43:** 129-135.

Handy, J. and others 2024. Antarctic benthic diatoms after 10 months of dark exposure: consequences for photosynthesis and cellular integrity. Frontiers in Plant Science **15:** 1326375.

Kaeriyama, H. and others 2011. Effects of temperature and irradiance on growth of strains belonging to seven Skeletonema species isolated from Dokai Bay, southern Japan. European journal of phycology **46:** 113-124.

Li, H., T. Xu, J. Ma, F. Li, and J. Xu. 2021. Physiological responses of *Skeletonema costatum* to the interactions of seawater acidification and the combination of photoperiod and temperature. Biogeosciences **18:** 1439-1449.

McCabe, R. M. and others 2016. An unprecedented coastwide toxic algal bloom linked to anomalous ocean conditions. Geophysical Research Letters **43:** 10,366-310,376.

McQuoid, M. R., and L. A. Hobson. 1996. Diatom resting stages. Journal of Phycology **32:** 889-902.

Montresor, M., C. Di Prisco, D. Sarno, F. Margiotta, and A. Zingone. 2013. Diversity and germination patterns of diatom resting stages at a coastal Mediterranean site. Marine Ecology Progress Series **484:** 79-95.

Pednekar, S. M., S. S. Bates, V. Kerkar, and S. P. Matondkar. 2018. Environmental factors affecting the distribution of *Pseudo-nitzschia* in two monsoonal estuaries of Western India and effects of salinity on growth and domoic acid production by *P. pungens*. Estuaries and Coasts **41:** 1448-1462.
