# Supplementary material for: The duration of nutrient limiting conditions can contribute to shaping subsequent diatom community composition: Insights from laboratory experiments
Source: PLoS One. 2026 Jul 21;21(7):e0333868. doi: 10.1371/journal.pone.0333868 (PMC13387546; doi:10.1371/journal.pone.0333868)
Supplement: Attachment — Submitted filename: Response to Reviewers.docx [file pone.0333868.s009.docx]

We thank the academic editor and all the reviewers for their time and expertise to review the manuscript. To facilitate the review of our revisions, changes made in response to Reviewer #2 comments are highlighted in blue, and changes made in response to Reviewer #3 comments are highlighted in grey throughout the revised manuscript (Revise Manuscript with Track Changes file).

Reviewer #2

**1. “Using the stationary phase as a proxy for nutrient limitation does not identify the specific limiting nutrient or exclude the possibility of co-limitation. As a result, observed growth differences cannot be linked to particular physiological mechanisms.”**

We agree with this comment. We have added a clarifying statement to the manuscript acknowledging that the use of stationary phase as a proxy for nutrient depletion does not allow identification of the specific limiting nutrient(s) or exclusion of co-limitation (L.145-L.149). We also highlight that the nutrient concentrations used in our growth media (GoM media) were considerably lower than those used in most standard culture media (e.g., F/2 or L1) (L.381-L.382), making nutrient depletion highly likely.

**2. “For Skeletonema costatum, the lack of growth recovery after prolonged limitation is a significant finding, but it should be interpreted with caution. The experimental design does not allow differentiation between cell death, resting spore formation, or reversible quiescent states*.*”**

We thank the reviewer for raising this important point. We have added clarifying statements in the Discussion (L.341-L344) sections acknowledging that the lack of growth recovery observed in S. costatum could reflect several alternative states, including cell death, resting spore formation, or a reversible quiescent state, and that our experimental design cannot distinguish among these possibilities.

**3. “Using non-axenic cultures adds complexity, as associated bacteria may affect nutrient dynamics and cellular recovery. This should be recognized as a source of variability rather than a strict limitation. In the conclusions section, it is stated that 'the laboratory setup differs in many aspects from that of ocean waters.' However, the conclusion does not specify whether the results are based on axenic or non-axenic cultures. I recommend clarifying this point in the conclusions.”**

We agree with this suggestion. We have revised the Conclusions section to explicitly state that experiments were conducted using non-axenic cultures, and we acknowledge that associated bacterial communities may have contributed some variability to the observed results (L.425-L.426). Following the reviewer's suggestion, we have framed this as a natural source of variability rather than a strict experimental limitation.

Reviewer #3

**1. “Consistency in species name abbreviations: The name of diatom is written in full the first time it is mentioned (e.g., Pseudo-nitzschia pungens), and then the short name is given. But starting with L. 252, sometimes the full name is used (L. 276, 328), sometimes the short name.”**

We thank the reviewer for this careful observation. We have reviewed the entire manuscript and standardized all species name abbreviations. Each species is now written in full at first mention, and the abbreviated form (e.g., P. pungens, P. australis, and S. costatum) is used consistently throughout the remainder of the text, including figure and table captions.

**2. “Abbreviation consistency for Specific Growth Rate (SGR): If you provide an abbreviation for the expression (Specific growth rates (SGR)), it should be used throughout the entire text. The text uses both the abbreviation and the expression itself (L. 235, 328, 331, 335, 337).”**

We have standardized the use of abbreviation “SGR” for specific growth rate and “LT” for Lag time throughout the manuscript, including figure and table titles. The abbreviation is now defined at first use and applied consistently thereafter.

**3. “In L. 128, 129, and 135, the abbreviation NL appears, and only in L. 165 is an explanation given of what this abbreviation stands for. This should be done at the first mention.”**

We thank reviewer for catching this. The abbreviation of “NL” (nutrient limitation) is now defined at first occurrence and used consistently thereafter.

**4. “I also suggest moving the table with the results for Experiment 2 from the 'Supporting Information' section to the article's body. These are the key findings of the study; they are used in the discussion and form the basis for the conclusions.”**

We agreed that the Experiment 2 results table is the central to the manuscript findings and appreciate this suggestion. We have moved it to main body of the manuscript as Table 2 (L.275).

**5. “I also propose removing the sentence 'Future research should study...' (L. 403–406) from the conclusion. It diminishes the significance of the obtained results and, at first glance, makes the study appear incomplete.”**

We agree with this suggestion. The sentence at L.403-406 has been removed from the Conclusion section. The Conclusion now ends on positive note that highlights the significance and broader implications of the findings.

**6. “In my very subjective opinion, Figure 1 is too big and uninformative. If it were reduced slightly, Figure S1 could be added from the 'Supporting Information', which is more useful for understanding the study results.”**

We appreciate this suggestion. We have reduced the size of Figure 1 and incorporated the former Figure S1 into Figure 1.
