# Supplementary material for: The duration of nutrient limiting conditions can contribute to shaping subsequent diatom community composition: Insights from laboratory experiments
Source: PLoS One. 2026 Jul 21;21(7):e0333868. doi: 10.1371/journal.pone.0333868 (PMC13387546; doi:10.1371/journal.pone.0333868)
Supplement: Peer review history

# Peer review: Decision Letter 0

**DOI:** 10.1371/journal.pone.0333868.r001

**Authors:** Rajdeep Roy

10 Dec 2025

PONE-D-25-51262

The duration of nutrient limiting conditions can contribute to shaping subsequent diatom community composition; insights from laboratory experiments

PLOS One

Dear Dr. Seto,

Thank you for submitting your manuscript to PLOS ONE. After careful consideration, we have decided that your manuscript does not meet our criteria for publication and must therefore be rejected.

Specifically:

==============================

There are several shortcomings out of which I only list few of them. The authors are encouraged to be more careful while doing such experiments. My comments are below. The authors suggest nutrient depletion from 16 µmol of nitrate and silicate to < 2 µmol within 5 days and then culture remains in that condition for next 12 days. This is very intriguing how only ambient temperature support growth for next 2 weeks without any nutrients? Does that happen in natural environment? The data presented do not show much difference between initial growth phase and days thereafter under nutrient deplete condition. This cannot happen! Please look at Table S1. For *P. pungens*  at temperature 9 °C the initial SGR is 0.36 after short NL at same temperature the growth rates are increased to 0.71 (How?). There are several artifacts such as this within the current manuscript. Even, some of the growth rates measured are higher than measured growth rates of Diatoms (See some of the publications by Victor Smetecek and others from natural environment and experimental set up). Why the cell counts were not plotted vs days vs various nutrients with time?.  The growth rate of same species (*P. pungens* ) for experiment one for initial replete is 1.58 then short NL is 1.24 which increases to 1.44 for 16 days NL (Table 1). How the diatoms surviving without any nutrients after 5 days ?.

I am sorry that we cannot be more positive on this occasion, but hope that you appreciate the reasons for this decision.

Kind regards,

Rajdeep Roy

Academic Editor

PLOS One

[Note: HTML markup is below. Please do not edit.]

Reviewers' comments:

Reviewer's Responses to Questions

**Comments to the Author**

1. Is the manuscript technically sound, and do the data support the conclusions?

The manuscript must describe a technically sound piece of scientific research with data that supports the conclusions. Experiments must have been conducted rigorously, with appropriate controls, replication, and sample sizes. The conclusions must be drawn appropriately based on the data presented.

Reviewer #1: Yes

**********

2. Has the statistical analysis been performed appropriately and rigorously?

Reviewer #1: Yes

**********

3. Have the authors made all data underlying the findings in their manuscript fully available?

The PLOS Data policy requires authors to make all data underlying the findings described in their manuscript fully available without restriction, with rare exception (please refer to the Data Availability Statement in the manuscript PDF file). The data should be provided as part of the manuscript or its supporting information, or deposited to a public repository. For example, in addition to summary statistics, the data points behind means, medians and variance measures should be available. If there are restrictions on publicly sharing data—e.g. participant privacy or use of data from a third party—those must be specified.

Reviewer #1: Yes

**********

4. Is the manuscript presented in an intelligible fashion and written in standard English?

PLOS ONE does not copyedit accepted manuscripts, so the language in submitted articles must be clear, correct, and unambiguous. Any typographical or grammatical errors should be corrected at revision, so please note any specific errors here.

Reviewer #1: Yes

**********

5. Review Comments to the Author

Please use the space provided to explain your answers to the questions above. You may also include additional comments for the author, including concerns about dual publication, research ethics, or publication ethics. (Please upload your review as an attachment if it exceeds 20,000 characters)

Reviewer #1: This research is fascinating, I’m happy to have the opportunity to review. It’s an excellent manuscript that contributes meaningfully to our understanding of diatom ecology and nutrient limitation effects, great work!

I’m not a diatom specialist, but the methods appear sound and well described. The results and discussion are clearly presented, and the study provides valuable insights into species-specific responses to nutrient stress — highly relevant to ongoing climate and ocean research, particularly in high-latitude systems.

For broader context, the authors might consider citing Barnard et al. for proxy data on high-latitude diatom responses to climate change.

Minor notes:

Typos on lines 343 and 355.

For future work, it would be great to see this extended through combined laboratory and field experiments to assess community-level dynamics and validate the lab findings in the field.

Overall, this is a well-written paper that is a very valuable contribution! Thanks :)

**********

6. PLOS authors have the option to publish the peer review history of their article (what does this mean?). If published, this will include your full peer review and any attached files.

If you choose “no”, your identity will remain anonymous but your review may still be made public.

**Do you want your identity to be public for this peer review?**  For information about this choice, including consent withdrawal, please see our Privacy Policy.

Reviewer #1: No

**********

[NOTE: If reviewer comments were submitted as an attachment file, they will be attached to this email and accessible via the submission site. Please log into your account, locate the manuscript record, and check for the action link "View Attachments". If this link does not appear, there are no attachment files.]

- - - - -

For journal use only: PONEDEC3

# Author comment: Author response to Decision Letter 1

**DOI:** 10.1371/journal.pone.0333868.r002

17 Feb 2026

December 22, 2025

Dr. Rajdeep Roy

PLOS One Academic Editor

Re: The duration of nutrient limiting conditions can contribute to shaping subsequent diatom community composition; insight from laboratory experiments

Dear Dr. Roy,

We would like to thank you for the time you have taken to handle our paper and provide your feedback. We were surprised, however, by your decision to reject the manuscript given the very positive feedback from reviewer 1, who stated that “This research is fascinating, I’m happy to have the opportunity to review. It’s an excellent manuscript that contributes meaningfully to our understanding of diatom ecology and nutrient limitation effects, great work!”. In your evaluation, we believe there is some misunderstanding about the structure of the experiments, which may require some text revision to clarify what was done. We explain using your comments.

1. “The authors suggest nutrient depletion from 16 µmol of nitrate and silicate to < 2 µmol within 5 days and then culture remains in that condition for next 12 days. This is very intriguing how only ambient temperature support growth for next 2 weeks without any nutrients”

You are correct that there would be no growth after the nutrients were consumed, and indeed that is the case. As we state in the manuscript (line 138), the cultures reached a stationary phase after 5 days, the stage where cell growth ceases but cells do not die. We go on to explain that cells remained in stationary phase for the following 12 days. Due to space considerations, we did not include a figure of cell numbers/abundance over time, but perhaps it would be helpful to include this graph as a supplementary figure.

We note that this behavior (growth followed by an extended period of senescence), is very common with phytoplankton species. Indeed diatom cultures are known to survive in stationary phase for weeks (e.g., Berges and Falkowski 1998; Handy et al. 2024, and see below).The mechanisms underlying this behavior, and the potential for subsequent recovery of growth, are little understood and is a key focus of this study.

The cells in our experiments resumed growth only when nutrients were re-introduced after this 12-day period of stationary phase, ‘mimicking’, for example, the re-introduction of nutrients to nutrient-depleted surface waters by vertical mixing. This response also is not unexpected, and indeed all three cultures showed robust growth after nutrient introduction after this “short” period of senescence. What is unique in our study, according to our literature searches, is that we tested how effectively growth re-initiates after a much longer period in stationary phase (27 days), and whether there were differences in this response among the three diatom species studied. The robust response of two, but not the other, is the important finding here; one which conceptually may explain why blooms comprise different diatoms at different times; a major conundrum in biological oceanography.

2. “The data presented do not show much difference between initial growth phase and days thereafter under nutrient deplete condition. This cannot happen!”

This misunderstanding stems from the misconception above. The experiments did not compare the initial growth rates with growth under the nutrient deplete (stationary phase) conditions. We compare instead the initial growth rates with those immediately after the nutrient ‘starved’ cells in the stationary phase were transferred to new fresh media or received a new injection of nutrients (i.e. after 12 or 27 days of nutrient depletion). The results show that when nutrients are resupplied after short ‘starvations’ (12 days) all three species resumed growth similar to that observed initially, but when experiencing a longer-term ‘starvations’ (27 days) the Skeletonema sp. did not grow upon the re-introduction of nutrients but the Pseudo-nitzschia spp. responded as if there had been no nutrient stress at all (i.e., growth rates and lag times were very similar to the growth response on Day 0 (i.e., with cells taken from rapidly growing seed cultures). In other words, some species of the Pseudo-nitzschia genus (tested here) can cease to grow when starved for nutrients but their metabolic ‘machinery’ is ready to capitalize on nutrients as soon as they become available. In contrast, the Skeletonema sp. here lost this ability and would require more time to recover. This difference is highly relevant to understanding the relative global success of Pseudo-nitzschia species.

3. “Even, some of the growth rates measured are higher than measured growth rates of Diatoms (See some of the publications by Victor Smetecek and others from natural environment and experimental set up)”

You are correct that the diatom growth rates here are higher than in some other studies, and relative to some estimates of in-situ rates, however, the growth rates in our experiments are not unusual. There are many publications showing diatom growth rates in laboratory cultures being > 1 per day, and indeed the growth rates in our experiments lie within the range reported in the literature for laboratory cultures of Pseudo-nitzschia (e.g., McCabe et al. 2016; Pednekar et al. 2018) and Skeletonema (e.g., Kaeriyama et al. 2011; Li et al. 2021). It is more difficult to directly compare growth rates under “optimal” laboratory conditions with those in-situ, where non-nutrient growth limitations influence the production and accumulation rates of cells (e.g., light limitation due to shorter days and mixing to depth, and grazing pressures that limit cell accumulation rates). Even so, the growth rates measured in these experiments also fall with the range of those observed for phytoplankton in natural marine water (e.g., the excellent review paper of Laws 2013. Evaluation of in situ phytoplankton growth rates: a synthesis of data from varied approaches” Annu. Rev. Mar. Sci.5:247-68). So, there is nothing suspect about the growth rates we report in our findings.

On the other side of things, we realize that we did make an error with the data provided in Supplementary material table 1 (S1_Table). Especially, the growth rate values for Pseudo-nitzschia pungens under short-term nutrient limitation at 9°C and 12°C were inadvertently swapped due to an input error in the R script used for the statistical analysis. We appreciate the editor’s careful attention in identifying this issue. The growth rate values shown in the main manuscript figures are correct and unaffected, as they generated independently from the original Excel dataset rather than the R script output. Further, we made only minor adjustment to the results section after this reanalysis, which can be tracked from the track change. To ensure full transparency and facilitate reanalysis, we have now provided all raw growth rate data in the supplementary materials (S3_Table). Additionally, as we note above, we probably also should have provided a supplementary figure showing cell abundance over time in the cultures, which would help clarify the culture behavior between the nutrient re-introductions (S5_Fig). However, we do not believe this presentation issues negate the importance of the work, as interpreted by Reviewer 1, or should result in outright rejection of the manuscript for publication. We respectfully request that the editor re-examine this decision, and we welcome their help to ensure that we optimize the revisions.

Sincerely,

Drajad Seto

(on behalf of all authors).

References

Berges, J. A., and P. G. Falkowski. 1998. Physiological stress and cell death in marine phytoplankton: induction of proteases in response to nitrogen or light limitation. Limnology and Oceanography 43: 129-135.

Handy, J. and others 2024. Antarctic benthic diatoms after 10 months of dark exposure: consequences for photosynthesis and cellular integrity. Frontiers in Plant Science 15: 1326375.

Kaeriyama, H. and others 2011. Effects of temperature and irradiance on growth of strains belonging to seven Skeletonema species isolated from Dokai Bay, southern Japan. European journal of phycology 46: 113-124.

Li, H., T. Xu, J. Ma, F. Li, and J. Xu. 2021. Physiological responses of Skeletonema costatum to the interactions of seawater acidification and the combination of photoperiod and temperature. Biogeosciences 18: 1439-1449.

McCabe, R. M. and others 2016. An unprecedented coastwide toxic algal bloom linked to anomalous ocean conditions. Geophysical Research Letters 43: 10,366-310,376.

McQuoid, M. R., and L. A. Hobson. 1996. Diatom resting stages. Journal of Phycology 32: 889-902.

Montresor, M., C. Di Prisco, D. Sarno, F. Margiotta, and A. Zingone. 2013. Diversity and germination patterns of diatom resting stages at a coastal Mediterranean site. Marine Ecology Progress Series 484: 79-95.

Pednekar, S. M., S. S. Bates, V. Kerkar, and S. P. Matondkar. 2018. Environmental factors affecting the distribution of Pseudo-nitzschia in two monsoonal estuaries of Western India and effects of salinity on growth and domoic acid production by P. pungens. Estuaries and Coasts 41: 1448-1462.

Attachment Submitted filename: Response to reviewer (academic editor)_Seto et al.docx

# Peer review: Decision Letter 1

**DOI:** 10.1371/journal.pone.0333868.r003

**Authors:** Rajdeep Roy

8 May 2026

PONE-D-25-51262R1

The duration of nutrient limiting conditions can contribute to shaping subsequent diatom community composition; insights from laboratory experiments

PLOS One

Dear Dr. Seto,

Thank you for submitting your manuscript to PLOS ONE. After careful consideration, we feel that it has merit but does not fully meet PLOS ONE’s publication criteria as it currently stands. Therefore, we invite you to submit a revised version of the manuscript that addresses the points raised during the review process.

==============================

Please submit your revised manuscript by Jun 22 2026 11:59PM. If you will need more time than this to complete your revisions, please reply to this message or contact the journal office at plosone@plos.org. When you're ready to submit your revision, log on to https://www.editorialmanager.com/pone/ and select the 'Submissions Needing Revision' folder to locate your manuscript file.

Please include the following items when submitting your revised manuscript:

- A letter that responds to each point raised by the academic editor and reviewer(s). You should upload this letter as a separate file labeled 'Response to Reviewers'.
- A marked-up copy of your manuscript that highlights changes made to the original version. You should upload this as a separate file labeled 'Revised Manuscript with Track Changes'.
- An unmarked version of your revised paper without tracked changes. You should upload this as a separate file labeled 'Manuscript'.

If you would like to make changes to your financial disclosure, please include your updated statement in your cover letter. Guidelines for resubmitting your figure files are available below the reviewer comments at the end of this letter.

If applicable, we recommend that you deposit your laboratory protocols in protocols.io to enhance the reproducibility of your results. Protocols.io assigns your protocol its own identifier (DOI) so that it can be cited independently in the future. For instructions see: https://journals.plos.org/plosone/s/submission-guidelines#loc-laboratory-protocols. Additionally, PLOS ONE offers an option for publishing peer-reviewed Lab Protocol articles, which describe protocols hosted on protocols.io. Read more information on sharing protocols at https://plos.org/protocols?utm_medium=editorial-email&utm_source=authorletters&utm_campaign=protocols.

As the corresponding author, your ORCID iD is verified in the submission system and will appear in the published article. PLOS supports the use of ORCID, and we encourage all coauthors to register for an ORCID iD and use it as well. Please encourage your coauthors to verify their ORCID iD within the submission system before final acceptance, as unverified ORCID iDs will not appear in the published article. *Only*  the individual author can complete the verification step; PLOS staff *cannot*  verify ORCID iDs on behalf of authors.

We look forward to receiving your revised manuscript.

Kind regards,

Rajdeep Roy

Academic Editor

PLOS One

Journal Requirements:

When submitting your revision, we need you to address these additional requirements.

1. Please ensure that your manuscript meets PLOS ONE's style requirements, including those for file naming. The PLOS ONE style templates can be found at

https://journals.plos.org/plosone/s/file?id=wjVg/PLOSOne_formatting_sample_main_body.pdf and

https://journals.plos.org/plosone/s/file?id=ba62/PLOSOne_formatting_sample_title_authors_affiliations.pdf

2. Thank you for stating the following financial disclosure:

“This research has been funded by National Science Foundation grant number OCE-1830029 for Dr. Mark L. Wells.”

Please state what role the funders took in the study. If the funders had no role, please state: "The funders had no role in study design, data collection and analysis, decision to publish, or preparation of the manuscript."

If this statement is not correct you must amend it as needed.

Please include this amended Role of Funder statement in your cover letter; we will change the online submission form on your behalf.

3.Please amend your manuscript to include a reference list. References must be placed at the end of the manuscript and numbered in the order that they appear in the text. For more information on the formatting of references, please visit the author guidelines at: http://journals.plos.org/plosone/s/submission-guidelines#loc-reference-style

If the reviewer comments include a recommendation to cite specific previously published works, please review and evaluate these publications to determine whether they are relevant and should be cited. There is no requirement to cite these works unless the editor has indicated otherwise.

Please review your reference list to ensure that it is complete and correct. If you have cited papers that have been retracted, please include the rationale for doing so in the manuscript text, or remove these references and replace them with relevant current references. Any changes to the reference list should be mentioned in the rebuttal letter that accompanies your revised manuscript. If you need to cite a retracted article, indicate the article’s retracted status in the References list and also include a citation and full reference for the retraction notice.

Additional Editor Comments (if provided):

[Note: HTML markup is below. Please do not edit.]

Reviewers' comments:

Reviewer's Responses to Questions

**Comments to the Author**

1. If the authors have adequately addressed your comments raised in a previous round of review and you feel that this manuscript is now acceptable for publication, you may indicate that here to bypass the “Comments to the Author” section, enter your conflict of interest statement in the “Confidential to Editor” section, and submit your "Accept" recommendation.

Reviewer #2: All comments have been addressed

Reviewer #3: (No Response)

**********

2. Is the manuscript technically sound, and do the data support the conclusions?

The manuscript must describe a technically sound piece of scientific research with data that supports the conclusions. Experiments must have been conducted rigorously, with appropriate controls, replication, and sample sizes. The conclusions must be drawn appropriately based on the data presented.

Reviewer #2: Yes

Reviewer #3: Yes

**********

3. Has the statistical analysis been performed appropriately and rigorously?

Reviewer #2: Yes

Reviewer #3: Yes

**********

4. Have the authors made all data underlying the findings in their manuscript fully available?

The PLOS Data policy requires authors to make all data underlying the findings described in their manuscript fully available without restriction, with rare exception (please refer to the Data Availability Statement in the manuscript PDF file). The data should be provided as part of the manuscript or its supporting information, or deposited to a public repository. For example, in addition to summary statistics, the data points behind means, medians and variance measures should be available. If there are restrictions on publicly sharing data—e.g. participant privacy or use of data from a third party—those must be specified.

Reviewer #2: Yes

Reviewer #3: Yes

**********

5. Is the manuscript presented in an intelligible fashion and written in standard English?

PLOS ONE does not copyedit accepted manuscripts, so the language in submitted articles must be clear, correct, and unambiguous. Any typographical or grammatical errors should be corrected at revision, so please note any specific errors here.

Reviewer #2: Yes

Reviewer #3: Yes

**********

6. Review Comments to the Author

Please use the space provided to explain your answers to the questions above. You may also include additional comments for the author, including concerns about dual publication, research ethics, or publication ethics. (Please upload your review as an attachment if it exceeds 20,000 characters)

Reviewer #2: The experimental design is robust, as it isolates key variables such as temperature, duration of nutritional stress, and taxonomic identity. This approach enables a comparative evaluation of diatom species responses under controlled conditions. Including both lag time and specific growth rate is a valuable addition, as it captures not only growth magnitude but also recovery capacity after periods of limitation.

However, some methodological considerations should be noted. Using the stationary phase as a proxy for nutrient limitation does not identify the specific limiting nutrient or exclude the possibility of co-limitation. As a result, observed growth differences cannot be linked to particular physiological mechanisms.

The study’s focus on integrative variables such as growth rate and lag time aligns with its broad experimental design, which includes multiple temperatures and stress durations.

The results indicate that Pseudo-nitzschia species, including Pseudo-nitzschia pungens and Pseudo-nitzschia australis, show a greater capacity for recovery after prolonged nutrient limitation compared to Skeletonema costatum.

For Skeletonema costatum, the lack of growth recovery after prolonged limitation is a significant finding, but it should be interpreted with caution. The experimental design does not allow differentiation between cell death, resting spore formation, or reversible quiescent states.

Similarly, using non-axenic cultures adds complexity, as associated bacteria may affect nutrient dynamics and cellular recovery. This should be recognized as a source of variability rather than a strict limitation.

In the conclusions section, it is stated that “the laboratory setup differs in many aspects from that of ocean waters.” However, the conclusion does not specify whether the results are based on axenic or non-axenic cultures. I recommend clarifying this point in the conclusions.

In summary, the study presents consistent, statistically supported results and is suitable for publication, provided the conclusions remain within the scope of the experimental conditions. The findings clearly show that the duration of nutrient-limiting stress influences diatom growth recovery, offering a valuable contribution to comparative ecophysiology. While the mechanistic scope is limited, the results provide a foundation for understanding species responses to prolonged nutrient scarcity. This contribution is timely, as it highlights potential implications for phytoplankton succession, especially under increased stratification, marine heatwaves, and climate change. The study does not address all questions but opens promising avenues for future research.

Reviewer #3: The article “The duration of nutrient limiting conditions can contribute to shaping subsequent diatom community composition; insights from laboratory experiments” presents results a laboratory study to examine how diatom community respond to short-term and prolonged periods of nutrient limitation.

In my opinion, the conducted laboratory experiments are well described and sufficiently labor-intensive. The presented results seem interesting. An enough number of references to the literature on the topic are also provided.

I don't have any significant comments.

However, the writing style isn't so good, making it difficult to comprehend. Therefore, I only have comments about the article's formatting.

First, I propose maintaining consistency throughout the article:

- The name of diatom is written in full the first time it is mentioned (for example, Pseudo-nitzschia pungens), and then the short name is given in the text (P. pungens). And this is correct. But then, starting with L. 252, sometimes the full name is used (L. 276, 328), sometimes the short name. They should be led to the same spelling. This also applies to the names of other diatom species;

- If you provide an abbreviation for the expression (Specific growth rates (SGR)), it should be used throughout the entire text of the article. In my opinion, this includes the titles of figures and tables. However, the text of the article uses both the abbreviation and the expression itself. (L. 235, 328, 331, 335, 337);

- In L. 128, 129, and 135, the abbreviation NL appears, and only in L. 165 is an explanation given of what this abbreviation stands for. This should be done at the first mention.

These are the main concepts and terms used in this study, so they should be treated more carefully.

I also suggest moving the table with the results for Experiment 2 from the "Supporting Information" section to the article's body. I understand the table is large, but it could be reformatted. Divide into 3 blocks “P. pungens”, “P. australis”, “S. costatum”; place “Initial”, “Short NL” and “Prolonged NL” in one row in the table header, and leave only the temperature in the left column. It would be very useful in the main body of the article. These are the key findings of the study; they are used in the discussion and form the basis for the conclusions. So, I think, they should be moved to the article.

I also propose removing the sentence "Future research should study..." (L. 403-406) from the conclusion. It diminishes the significance of the obtained results and, at first glance, makes the study appear incomplete. Therefore, it would be better not to use it in the conclusion.

Also, in my very subjective opinion, Figure 1 is too big and uninformative. If it were reduced slightly, Figure S1 could be added from the "Supporting Information", which is more useful for understanding the study results.

In general, I appreciate the effort invested by the authors into this research. I propose to publish this article after corrections.

**********

7. PLOS authors have the option to publish the peer review history of their article (what does this mean?). If published, this will include your full peer review and any attached files.

If you choose “no”, your identity will remain anonymous but your review may still be made public.

**Do you want your identity to be public for this peer review?**  For information about this choice, including consent withdrawal, please see our Privacy Policy.

Reviewer #2: No

Reviewer #3: No

**********

[NOTE: If reviewer comments were submitted as an attachment file, they will be attached to this email and accessible via the submission site. Please log into your account, locate the manuscript record, and check for the action link "View Attachments". If this link does not appear, there are no attachment files.]

To ensure your figures meet our technical requirements, please review our figure guidelines: https://journals.plos.org/plosone/s/figures

You may also use PLOS’s free figure tool, NAAS, to help you prepare publication quality figures: https://journals.plos.org/plosone/s/figures#loc-tools-for-figure-preparation.

NAAS will assess whether your figures meet our technical requirements by comparing each figure against our figure specifications.

# Author comment: Author response to Decision Letter 2

**DOI:** 10.1371/journal.pone.0333868.r004

22 Jun 2026

We thank the academic editor and all the reviewers for their time and expertise to review the manuscript. To facilitate the review of our revisions, changes made in response to Reviewer #2 comments are highlighted in blue, and changes made in response to Reviewer #3 comments are highlighted in grey throughout the revised manuscript (Revise Manuscript with Track Changes file).

Reviewer #2

1. “Using the stationary phase as a proxy for nutrient limitation does not identify the specific limiting nutrient or exclude the possibility of co-limitation. As a result, observed growth differences cannot be linked to particular physiological mechanisms.”

We agree with this comment. We have added a clarifying statement to the manuscript acknowledging that the use of stationary phase as a proxy for nutrient depletion does not allow identification of the specific limiting nutrient(s) or exclusion of co-limitation (L.145-L.149). We also highlight that the nutrient concentrations used in our growth media (GoM media) were considerably lower than those used in most standard culture media (e.g., F/2 or L1) (L.381-L.382), making nutrient depletion highly likely.

2. “For Skeletonema costatum, the lack of growth recovery after prolonged limitation is a significant finding, but it should be interpreted with caution. The experimental design does not allow differentiation between cell death, resting spore formation, or reversible quiescent states.”

We thank the reviewer for raising this important point. We have added clarifying statements in the Discussion (L.341-L344) sections acknowledging that the lack of growth recovery observed in S. costatum could reflect several alternative states, including cell death, resting spore formation, or a reversible quiescent state, and that our experimental design cannot distinguish among these possibilities.

3. “Using non-axenic cultures adds complexity, as associated bacteria may affect nutrient dynamics and cellular recovery. This should be recognized as a source of variability rather than a strict limitation. In the conclusions section, it is stated that 'the laboratory setup differs in many aspects from that of ocean waters.' However, the conclusion does not specify whether the results are based on axenic or non-axenic cultures. I recommend clarifying this point in the conclusions.”

We agree with this suggestion. We have revised the Conclusions section to explicitly state that experiments were conducted using non-axenic cultures, and we acknowledge that associated bacterial communities may have contributed some variability to the observed results (L.425-L.426). Following the reviewer's suggestion, we have framed this as a natural source of variability rather than a strict experimental limitation.

Reviewer #3

1. “Consistency in species name abbreviations: The name of diatom is written in full the first time it is mentioned (e.g., Pseudo-nitzschia pungens), and then the short name is given. But starting with L. 252, sometimes the full name is used (L. 276, 328), sometimes the short name.”

We thank the reviewer for this careful observation. We have reviewed the entire manuscript and standardized all species name abbreviations. Each species is now written in full at first mention, and the abbreviated form (e.g., P. pungens, P. australis, and S. costatum) is used consistently throughout the remainder of the text, including figure and table captions.

2. “Abbreviation consistency for Specific Growth Rate (SGR): If you provide an abbreviation for the expression (Specific growth rates (SGR)), it should be used throughout the entire text. The text uses both the abbreviation and the expression itself (L. 235, 328, 331, 335, 337).”

We have standardized the use of abbreviation “SGR” for specific growth rate and “LT” for Lag time throughout the manuscript, including figure and table titles. The abbreviation is now defined at first use and applied consistently thereafter.

3. “In L. 128, 129, and 135, the abbreviation NL appears, and only in L. 165 is an explanation given of what this abbreviation stands for. This should be done at the first mention.”

We thank reviewer for catching this. The abbreviation of “NL” (nutrient limitation) is now defined at first occurrence and used consistently thereafter.

4. “I also suggest moving the table with the results for Experiment 2 from the 'Supporting Information' section to the article's body. These are the key findings of the study; they are used in the discussion and form the basis for the conclusions.”

We agreed that the Experiment 2 results table is the central to the manuscript findings and appreciate this suggestion. We have moved it to main body of the manuscript as Table 2 (L.275).

5. “I also propose removing the sentence 'Future research should study...' (L. 403–406) from the conclusion. It diminishes the significance of the obtained results and, at first glance, makes the study appear incomplete.”

We agree with this suggestion. The sentence at L.403-406 has been removed from the Conclusion section. The Conclusion now ends on positive note that highlights the significance and broader implications of the findings.

6. “In my very subjective opinion, Figure 1 is too big and uninformative. If it were reduced slightly, Figure S1 could be added from the 'Supporting Information', which is more useful for understanding the study results.”

We appreciate this suggestion. We have reduced the size of Figure 1 and incorporated the former Figure S1 into Figure 1.

Attachment Submitted filename: Response to Reviewers.docx

# Peer review: Decision Letter 2

**DOI:** 10.1371/journal.pone.0333868.r005

**Authors:** Rajdeep Roy

6 Jul 2026

The duration of nutrient limiting conditions can contribute to shaping subsequent diatom community composition; insights from laboratory experiments

PONE-D-25-51262R2

Dear Dr. Seto,

We’re pleased to inform you that your manuscript has been judged scientifically suitable for publication and will be formally accepted for publication once it meets all outstanding technical requirements.

Within one week, you’ll receive an e-mail detailing the required amendments. When these have been addressed, you’ll receive a formal acceptance letter and your manuscript will be scheduled for publication.

An invoice will be generated when your article is formally accepted. Please note, if your institution has a publishing partnership with PLOS and your article meets the relevant criteria, all or part of your publication costs will be covered. Please make sure your user information is up-to-date by logging into Editorial Manager at Editorial Manager® and clicking the ‘Update My Information' link at the top of the page. For questions related to billing, please contact billing support.

If your institution or institutions have a press office, please notify them about your upcoming paper to help maximize its impact. If they’ll be preparing press materials, please inform our press team as soon as possible -- no later than 48 hours after receiving the formal acceptance. Your manuscript will remain under strict press embargo until 2 pm Eastern Time on the date of publication. For more information, please contact onepress@plos.org.

Kind regards,

Rajdeep Roy

Academic Editor

PLOS One

Additional Editor Comments (optional):

Reviewers' comments:

Reviewer's Responses to Questions

**Comments to the Author**

1. If the authors have adequately addressed your comments raised in a previous round of review and you feel that this manuscript is now acceptable for publication, you may indicate that here to bypass the “Comments to the Author” section, enter your conflict of interest statement in the “Confidential to Editor” section, and submit your "Accept" recommendation.

Reviewer #2: All comments have been addressed

Reviewer #3: All comments have been addressed

**********

2. Is the manuscript technically sound, and do the data support the conclusions?

The manuscript must describe a technically sound piece of scientific research with data that supports the conclusions. Experiments must have been conducted rigorously, with appropriate controls, replication, and sample sizes. The conclusions must be drawn appropriately based on the data presented.

Reviewer #2: Yes

Reviewer #3: Yes

**********

3. Has the statistical analysis been performed appropriately and rigorously?

Reviewer #2: Yes

Reviewer #3: Yes

**********

4. Have the authors made all data underlying the findings in their manuscript fully available?

The PLOS Data policy requires authors to make all data underlying the findings described in their manuscript fully available without restriction, with rare exception (please refer to the Data Availability Statement in the manuscript PDF file). The data should be provided as part of the manuscript or its supporting information, or deposited to a public repository. For example, in addition to summary statistics, the data points behind means, medians and variance measures should be available. If there are restrictions on publicly sharing data—e.g. participant privacy or use of data from a third party—those must be specified.

Reviewer #2: Yes

Reviewer #3: Yes

**********

5. Is the manuscript presented in an intelligible fashion and written in standard English?

PLOS ONE does not copyedit accepted manuscripts, so the language in submitted articles must be clear, correct, and unambiguous. Any typographical or grammatical errors should be corrected at revision, so please note any specific errors here.

Reviewer #2: Yes

Reviewer #3: Yes

**********

6. Review Comments to the Author

Please use the space provided to explain your answers to the questions above. You may also include additional comments for the author, including concerns about dual publication, research ethics, or publication ethics. (Please upload your review as an attachment if it exceeds 20,000 characters)

Reviewer #2: (No Response)

Reviewer #3: (No Response)

**********

7. PLOS authors have the option to publish the peer review history of their article (what does this mean?). If published, this will include your full peer review and any attached files.

If you choose “no”, your identity will remain anonymous but your review may still be made public.

**Do you want your identity to be public for this peer review?**  For information about this choice, including consent withdrawal, please see our Privacy Policy.

Reviewer #2: No

Reviewer #3: No

**********

# Editor report: Acceptance letter

**DOI:** 10.1371/journal.pone.0333868.r006

**Authors:** Rajdeep Roy

PONE-D-25-51262R2

PLOS One

Dear Dr. Seto,

I'm pleased to inform you that your manuscript has been deemed suitable for publication in PLOS One. Congratulations! Your manuscript is now being handed over to our production team.

At this stage, our production department will prepare your paper for publication. This includes ensuring the following:

* All references, tables, and figures are properly cited

* All relevant supporting information is included in the manuscript submission,

* There are no issues that prevent the paper from being properly typeset

You will receive further instructions from the production team, including instructions on how to review your proof when it is ready. Please keep in mind that we are working through a large volume of accepted articles, so please give us a few days to review your paper and let you know the next and final steps.

Lastly, if your institution or institutions have a press office, please let them know about your upcoming paper now to help maximize its impact. If they'll be preparing press materials, please inform our press team within the next 48 hours. Your manuscript will remain under strict press embargo until 2 pm Eastern Time on the date of publication. For more information, please contact onepress@plos.org.

You will receive an invoice from PLOS for your publication fee after your manuscript has reached the completed accept phase. If you receive an email requesting payment before acceptance or for any other service, this may be a phishing scheme. Learn how to identify phishing emails and protect your accounts at https://explore.plos.org/phishing.

If we can help with anything else, please email us at customercare@plos.org.

Thank you for submitting your work to PLOS ONE and supporting open access.

Kind regards,

PLOS ONE Editorial Office Staff

on behalf of

Dr. Rajdeep Roy

Academic Editor

PLOS One
